# Past and Present Approaches to Diagnosis of Active Pulmonary Tuberculosis

**DOI:** 10.3389/fmed.2021.709793

**Published:** 2021-09-23

**Authors:** Anna Ritah Namuganga, Novel N. Chegou, Harriet Mayanja-Kizza

**Affiliations:** ^1^Uganda–Case Western Research Collaboration-Mulago, Kampala, Uganda; ^2^Joint Clinical Research Centre, Kampala, Uganda; ^3^College of Health Sciences, Makerere University, Kampala, Uganda; ^4^DSI-NRF Centre of Excellence for Biomedical Tuberculosis Research, South African Medical Research Council Centre for Tuberculosis Research, Division of Molecular Biology and Human Genetics, Faculty of Medicine and Health Sciences, Stellenbosch University, Cape Town, South Africa

**Keywords:** tuberculosis, diagnosis, biomarkers, immunodiagnostic assay, immune response

## Abstract

Tuberculosis disease continues to contribute to the mortality burden globally. Due to the several shortcomings of the available diagnostic methods, tuberculosis disease continues to spread. The difficulty to obtain sputum among the very ill patients and the children also affects the quick diagnosis of tuberculosis disease. These challenges warrant investigating different sample types that can provide results in a short time. Highlighted in this review are the approved pulmonary tuberculosis diagnostic methods and ongoing research to improve its diagnosis. We used the PRISMA guidelines for systematic reviews to search for studies that met the selection criteria for this review. In this review we found out that enormous biosignature research is ongoing to identify host biomarkers that can be used as predictors of active PTB disease. On top of this, more research was also being done to improve already existing diagnostic tests. Host markers required more optimization for use in different settings given their varying sensitivity and specificity in PTB endemic and non-endemic settings.

## Introduction

### Tuberculosis Burden and Eradication Strategies

Tuberculosis (TB) continues to infect many people worldwide causing at least 1.2 million deaths annually. Reducing new infections and mortality among patients who are difficult to diagnose with the currently available methods is paramount (1). Improved, timely and cost-effective diagnosis of active TB disease is a key strategy for ending the TB epidermic.

The World Health Organization (WHO) implemented strategies to end TB since 1995 to date (2, 3) focused on the sustainable development goal 3, 3.3 whose main objective is to end pandemics like TB by 2030. The SDG 3.3.2 aims at reducing TB incidence in the population (4). Achieving all these strategies is highly dependent on the widespread diagnosis of pulmonary TB cases within the population (5).

Given that immune response against TB is a continuum, predicting those who are progressing from “latent tuberculosis infection (LTBI)” to active TB disease is as important in the diagnosis of TB disease among those who may require preventive therapy (6) including the HIV co-infected subjects. While tests such as “Interferon-gamma release assays (IGRAs)” can indicate that an individual was exposed to *Mtb*, owing to T cell reactivity to TB antigens, these have not shown good results in diagnosing active pulmonary TB disease. Antibody tests on the other hand are not approved for TB diagnosis by WHO due to their poor sensitivity and specificity (4).

Current diagnostic methods have limitations like long turnaround time, low sensitivity, cost, inability to distinguish active TB disease from latent infection, high infrastructure, and training needs among others (7). Molecular diagnostic tests are currently providing a rapid TB diagnostic platform providing results within 2 h thereby improving patient care (8). Currently, the Gene Xpert test is widely distributed in resource-limited settings for TB disease screening in high TB endemic settings (9).

Although we have good tests for tuberculosis, high TB incidence could still be attributed to challenges with the available diagnostic tests as well as the inability to diagnose TB disease early. The very young (10), the elderly who present as pneumonia and HIV infected persons on antibiotics usually present with paucibacillary TB, negative smear thus missed diagnosis (11). The delay in seeking health services as well as poor health services delivery also contributes to an increase in TB incidence. In resource-limited settings, several factors contribute to high TB incidence. These include, the delay in seeking health services and long turnaround time (TAT) for reading smear slides given the high workload in the health centers (12). There is a drive to enhance research using readily available samples to identify bio-signatures that can diagnose active TB disease.

In many endemic and often resource-limited settings, the diagnosis of active TB disease is a challenge despite the important advances in the development of new diagnostic tools. For this reason, a simple, sensitive, and rapid diagnostic point of care (POC) test may be required for screening of individuals presumed to have active TB in high TB endemic settings as maybe offered by immunological tests. These can then be followed by confirmatory tests as required. To date, despite all research efforts, none of the immunologically based TB disease detection tests is WHO approved (13).

In this review, we highlight the approved diagnostic methods (Table 1) for TB and discuss attempts made to identify blood-based diagnostic approaches and other potential samples which may lead to a point of care test for TB. Also highlighted are other diseases that may affect pulmonary tuberculosis disease diagnosis and progression.

**Table 1:** Currently approved tuberculosis diagnostics.

**Diagnostic method**	**Advantages**	**Limitations**
Chest radiography	- Can be used as an initial screening tool	- Costly - Not widely accessible - test accuracy may be affected by variation between readers.
Direct microscopic examination^*^: identifies acid-fast bacilli through the use of Ziehl–Neelsen staining	- Fast - low cost	- Low sensitivity, - Unable to discriminate between mycobacterial species, - Time-consuming for lab technicians, - Depends on sputum sample quality and bacterial load, - Cannot detect drug resistance - Cannot differentiate live or dead bacilli
Fluorescence light-emitting diode microscopy: Identifies acid-fast bacilli stained with auramine, a fluorescent dye.	- Fast - Improved sensitivity	- High cost, - Needs well-trained technicians, - Poor performance among the HIV positive who are paucibacillary, - Depends on the quality and bacterial load of the sputum specimen - Cannot detect drug resistance - Cannot differentiate live or dead bacilli
Culture (liquid): Identifies *Mtb* with radio-active and fluorometric growth indicators (MGIT),	- High sensitivity - Permits testing for drug resistance.	- Expensive, - Needs well-trained technicians, - Needs established infrastructure like Biosafety facilities
Culture (Solid): Identifies *Mtb* growth on solid (LJ and Middlebrook) media	- Permits testing for drug resistance.	- Long turnaround time, - Low sensitivity - Expensive, - Needs well-trained technicians, - Needs established infrastructure like Biosafety facilities
Xpert MTB/RIF^*^: detect *Mtb* complex DNA and mutations associated with rifampicin resistance	- High sensitivity, - Permits testing for rifampicin resistance, - Low turn around time (2 h)	- High cost for procuring the machine and consumables like cartridges - Needs well-trained technicians, - Cannot differentiate live or dead bacilli - Has stringent operational requirements like uninterrupted power supply, temperature below 30°C, adequate storage for cartridges at temperatures below 28°C - Has programmatic requirements like software and annual calibration, - Waste disposal for cartridges is a concern
TB LAM^*^: Detects lipopolysaccharide which is shed from mycobacterial cell walls in urine.	- Easy to collect and store, - Does not require high biosafety requirements. - Has improved sensitivity among HIV positive	- Applicable primarily among HIV/AIDs patients - Sub-optimal sensitivity

*Xpert MTB RIF, GeneXpert MTB/RIF assay (Cepheid, Sunnyvale, CA); LAM, lipoarabinomannan; MGIT, Mycobacterium growth indicator tube; LJ, Löwenstein-Jensen*.

* *Applies to resource-limited settings, which often have the highest TB burdens*.

## Research Question and Aims

What are the past and present approaches to diagnosis of active pulmonary tuberculosis?

### Objectives

1. To highlight the currently approved active PTB diagnostic methods.
2. To describe the advances in blood biomarkers for diagnosis of TB disease in children and adults regardless of HIV status.
3. To describe the performance of *Mtb* derived markers for predicting active PTB disease in HIV-negative adolescents and adults.

### Methods

This review was done using the Preferred Reporting Items for Systematic Review and Meta-Analysis Protocols (PRISMA) checklist. A thorough literature search for studies that meet the inclusion criteria was done and the relevant data extracted. The study was conducted and completed between March 2020 and January 2021.

### Inclusion Criteria

The following studies were included in this review: cross-sectional, cohort, case–control, and randomized control studies involving active PTB subjects regardless of HIV status. Also, studies using host blood biomarker signatures to predict active PTB disease with a microbiological reference standard of either *Mtb* culture and/or Xpert MTB/RIF or smear microscopy, studies using either TST or IGRA for predicting active PTB disease. In addition, studies comparing TB disease cases to controls with or without other diseases, and with or without latent *Mtb* infection plus pediatric and adults PTB studies. Studies published both as abstracts and full articles between January 1995 to January 2021 published in English regardless of location or country of origin were also included.

### Exclusion

We excluded PTB treatment and latently *Mtb*-infected biomarker studies, conducted in animals; did not report sensitivity and specificity, and where authors did not respond to enquiries for data within 4 weeks of inquiry. Unpublished reports and conference proceedings were excluded due to absence of peer review and difficulty in obtaining data.

### Literature Search

The searches were conducted in Medline through PubMed and Google scholar databases. The search Key terms used included: Tuberculosis, *Mtb*, diagnosis, diagnostics, detect, predict, blood, immune markers, immunodiagnostic markers, host, TB human biomarker, TB diagnostic bio-signature, transcriptome, transcriptomic, *Mtb* RNA, accuracy, diagnostic accuracy, performance, sensitivity and specificity, area under the curve (AUC), receiver operating characteristic (ROC) curves. Any other citations from identified literature that were not originally identified during the search were also included.

### Study Selection

ARN searched the databases for all studies that met the selection criteria by title, abstract and later by full text. The selected studies were further screened by two reviewers (HMK and NC) for their quality as to whether they were fit for inclusion in this review. Articles were categorized as (i) included, (ii) not included, or (iii) pending reviewer discretion. The two reviewers conferred to resolve any disagreements about pending publications, and if a consensus could not be reached, a third reviewer (ARN) acted as the tiebreaker.

### Data Extraction and Management

**Figure 1:**
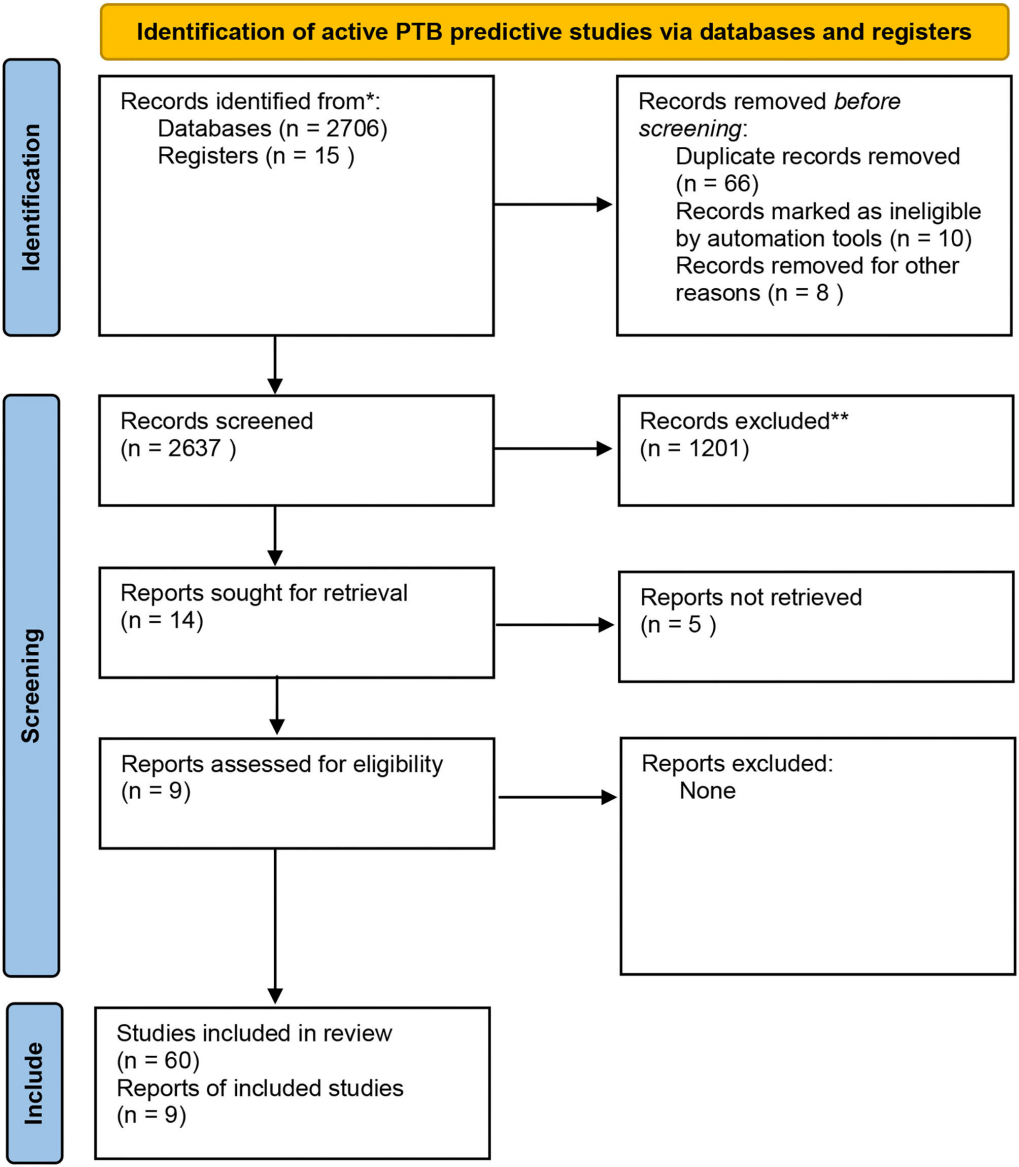
PRISMA flow chart indicating the data extraction process. Adapted from Page et al. (14).

Data was extracted from the relevant articles into a Microsoft word file which was shared among the reviewers to facilitate online sharing and screening of data between reviewers. For each study, we extracted the reported performance data; the diagnostic accuracy, sensitivity, specificity as per the flow chart attached (Figure 1).

## Results

### Current Diagnostic Methods

The currently approved methods for TB diagnosis are listed in Table 1 below with their limitations.

#### Sputum Exam

There are several currently approved methods for TB diagnosis and these are either microbiological or molecular methods that detect the pathogen itself or its products. Sputum smear microscopy and culture still present limitations such as low sensitivity (15). Microscopy has been advanced with fluorescence light-emitting diode which provides better sensitivity (6, 16). Sputum culture although more sensitive than sputum microscopy presents challenges of long TAT, high cost plus high infrastructure and training needs (17). Among those who show up in the TB clinic to be screened, delayed diagnosis of active pulmonary TB contributes to increased PTB disease burden and spread of the infection (18). All sputum-based diagnostic methods have limited use among children and the very sick who are unable to cough.

#### GeneXpert

Currently, the Xpert© MTB/RIF assay (Cepheid Inc, Sunnyvale, Calif., USA) is what comes close to a rapid molecular diagnostic tuberculosis test although it is dependent on good quality sputum sample (19). Tuberculosis diagnosis can also be through the detection of host biomarkers or pathogen biomarkers such as *Mycobacterium tuberculosis (Mtb)* products (20). These challenges lead to delayed diagnosis of active pulmonary TB thereby contributing to increased PTB disease burden and spread of infection.

#### Chest Imaging

Chest radiography (x-ray) has the potential as an initial screening test for active pulmonary TB. Imaging advances include Positron emission tomography (PET) scans which are showing potential for identifying active TB lesions. 2-[^18^F]fluoro-2-deoxyglucose (^18^F-FDG) PET/CT scans have shown potential for detecting active pulmonary TB lesions (21) and treatment response (22).

In cases where sputum is not available e.g., in children, attempts are made to collect other specimens e.g., gastric washes. The collection of these specimens is not always practical in resource-limited settings, which often do not have highly skilled health care workers. These and other specimens are collected from extra pulmonary TB patients (EPTB) for TB diagnosis but the yield of the microbiological tests is still low due to the paucibacillary nature of these specimens compared to sputum (23).

For this reason, blood-based diagnostic methods remain paramount for quick tuberculosis point of care tests.

### Immunodiagnostic Tests for Tuberculosis

#### Promising Blood-Based Diagnostic Approaches Under Research and Development

Tests making use of the immune responses to tuberculosis would be a valid addition to current tests to improve active tuberculosis diagnosis. Some immunological test formats will have the possibility of minimal invasiveness, minimal training requirements, simplicity, and low cost and will easily serve as POC tests for rapid diagnosis of tuberculosis (24). Some of these tests are available as immune-chromatographic methods as lateral flow strips and as “enzyme-linked immunosorbent assay (ELISA)” (25).

The absence of validated active TB predictive biomarkers hinders their use as immune-based active TB disease diagnostic methods. This coupled with the inability to differentiate disease states warrants continued research. Ideally, the most appropriate cytokine panel for studying PTB disease is one that covers the whole spectrum of TB infection and disease (26) which includes may include “T helper 1 and 2 (Th1/Th2)” balance, granuloma formation, macrophage activation, T-cell stimulation and limitation of inflammation (27). In this review, the following blood-based avenues have been discussed: LTBI blood-based tests as predictors of active TB disease, antibodies, cytokines, blood transcriptomic signatures, and immune metabolic markers.

#### Humoral Response

##### Antibody Tests for Active Tuberculosis

Antibodies to active TB have previously been used as both therapeutic (28) and diagnostic agents of TB (29–31). Antibody levels have shown potential as predictors of high bacterial load and active TB disease progression among the LTBI (32). *In-vitro* immunodiagnostic studies utilize *Mtb* antigens in single or multiple combinations to evaluate the production of “immunoglobulin (Ig)” A, G, and M antibodies in blood samples (33, 34). These are incorporated onto lateral flow strips that are easy to read on lateral flow readers (35). Serological tests that have been developed for the market so far (Table 2) include the Anda TB test, IBL test, Pathozyme-Myco IgG test (Myco G), and TB complex test (36). While these approaches have yielded promising results, there are no approved tests in the market yet due to their poor sensitivity and specificity ranging between 57 and 94% (37, 38).

**Table 2:** Currently available serological tests for TB diagnosis.

**Serological test**	**Antibody class**	**Sensitivity (%)**	**Specificity (%)**	**Antigen**
IBL	IgG	76	57	recombinant proteins 18, 36, and 40 kDa
Myco G	IgG	50	94	Lipoarabinomannan & Rv0934
Anda TB	IgG	78	79	Antigen 60
TB complex test	IgG	52	93	Rv0934 & Rv2031c

Other *Mtb* antigens that have been used include BCG, PPD (39, 40), LAM, TB15.3, TB51A, CFP10-ESAT6-A, CFP, CW (41, 42). Titers for IgG were higher in active TB disease compared with LTBI or controls in children (40) and adults (39, 41, 42). Higher IgG titers also correlated with reduced T cell counts and disease progression as was seen in TB/HIV) (42, 43).

A study done in South Africa investigated the serodiagnostic reactivity of periplasmic phosphate-binding lipoprotein PstS3 (Rv0928), L-alanine dehydrogenase (AlaDH) (Rv2780), nitrate/nitrite response transcriptional regulator NarL (Rv0844c), 19 kDa lipoprotein antigen pre- cursor LpqH (Rv3763), and lipoprotein MPT83 (Rv2873) evaluated IgG and IgA antibody production. High IgA levels but not IgG were observed among the LTBI group and were predictive of progression to active TB disease in this group (32). In another study from the same group that assessed IgA and G levels among slow and fast responders to TB treatment, slow responders had higher IgA and IgG levels compared to fast responders. IgG levels dropped significantly following TB treatment (44).

Despite being an attractive point of care test for active pulmonary tuberculosis, antibody tests for PTB have not been approved for use globally due to their inaccuracy, inconsistency, and poor specificity (13). This inaccuracy can be due to many factors including cross-reactivity (45), age, and the inability to differentiate recent antibodies from already existing ones. In high TB endemic areas, high background antibody concentrations among controls hinder the prognostic potential of antibodies among these populations. Given that all pathogens generate an antibody response, it is difficult to identify whether antibodies are toward a recent or a previously established active PTB disease *in vivo*.

Due to the unsatisfactory performance of sero-diagnostics, assays utilizing *Mtb*-specific antibodies (45) in biological samples like serum are available on the market but are not approved by WHO or “Food and drug authority (FDA).” In-depth studies need to be done to assess *Mtb* humoral response as well as the inability of antibodies to confer protective immunity to tuberculosis (46).

#### Cell-Mediated Immune Response to Tuberculosis

##### TST and IGRA for Active TB Diagnosis

The “tuberculin skin test (TST)” and “interferon-gamma release assays (IGRA)” are used globally to determine exposure to *Mtb* but are not ideal for predicting recent infection or active Tb disease, especially in high TB endemic areas. Although relatively old, the TST detects *Mtb* and *Mtb-*related purified protein derivatives owing to its low specificity as it contains a crude mixture of antigens, which are also available in “non-tuberculous mycobacterium NTMs.” Because TST has low sensitivity and specificity, the (IGRA) has been introduced to determine exposure to *Mtb* but not as a replacement for the TST. In the developed world, the IGRA is used as supplementary tests to TST for improved performance given the age dependant production of interferon-gamma, especially among pediatrics. Given that multiple cytokines and chemokines contribute to the immune response to the bacilli, a biomarker signature rather than a single cytokine or chemokine is required to predict active TB disease and infection (47).

A signature comprising, IL1ra, IFNg, MIP-1b, and TGF could predict active TB disease better in QuantiFERON (QFT) gold-in-tube supernatants (48). Another study evaluating additional markers in (QFT) gold plus supernatants (49) showed differential expression of GM-CSF, IL-33, ITAC-1, I-309, MIG, ADAMTS13, IL-1 α, IL-22, IL-2, IL-3, and TGF-α among those with active TB disease. Recently, efforts focused on the discovery of *ex vivo* biomarkers are taking lead as these may be more easily converted to point of care (POC) tests. Given the huge amount of research in this area, it is paramount to translate the researched signatures to POC tests. Studies have been done to evaluate the potential of TST and IGRA for active PTB diagnosis but these do not have the predictive potential for active PTB diagnosis (50, 51).

##### TB-Specific Antigen to Phytohaemagglutinin Ratio for Active PTB Diagnosis

The T-SPOT assay that analyses IFNg producing T Spot-forming cells (sfc) isolated from PBMCs for their response to TB antigens (Ag); secreted antigenic target 6 (ESAT-6) and culture filtrate protein 10 (CFP-10) and phytohaemagglutinin (PHA) TB Ags ratio to predict LTB infection. Among the immune compromised and children above 2 years, this assay has sensitivity and specificity >95%. Originally, the T-SPOT assay like all IGRAs was not suitable for active TB diagnosis due to its inability to differentiate active PTB from LTBI. Despite that shortcoming, PHA IFNg levels were higher in active PTB group compared to the no-TB group using ESAT-6 and CFP10 sfc ratio. Recently a modification that uses the TBAg/PHA ratio in T-SPOT.TB assay was analyzed for its ability to diagnose active PTB in a PTB endemic setting. A study by Wang et al. (52) proposed assesseing the ratio of ESAT-6 and CFP10 combined to PHA for its predictive potential of active PTB disease. The results showed good performance of the TB Ag/PHA with sensitivity of 82.1% and specificity of 90.7% to distinguish active PTB disease from LTBI. These results correlated with the results in a larger multi center study offering sensitivity and specificity above 80% (53). Among the immune compromised, the sensitivity reduced to 79.21 and specificity increased to 94.05 but was still high enough for use among this population (54). In order to improve sensitivity, modifications such as combining the TB Ag/PHA ratio with tests like Xpert MTB/RIF and Prealbumin have been made. When the Xpert MTB/RIF was used together with the TB Ag/PHA ratio to distinguish active PTB from LTBI, sensitivity improved to 88.05% and specificity of 96.26% (55) while with prealbumin sensitivity was 91.67% and specificity was 90.48% (56).

#### Host Inflammatory Biomarkers Detectable in *ex-vivo* Samples

##### Plasma and Serum Proinflammatory Cytokines, Chemokines, and Growth Factors

Recent advances use multiplex ELISAs to analyze concentrations of cytokines and chemokines for their ability to differentiate PTB patients from latently infected subjects or healthy controls. When serum and plasma levels of these markers were evaluated, pro-inflammatory cytokines and chemokines like VEGF (57), IFNg, IL-2, TNF alpha, IL-12p40, IL-10, GM-CSF, IL-1 beta, and IL-6 were higher among active PTB patients compared to healthy controls (58–60). Similarly, the same markers were higher among active PTB children than their healthy counterparts. Importantly, pediatric levels were lower than adult levels mainly because the adult immune system is more developed compared to the pediatric one (27). Active TB predictive biosignatures included; “IL-6, MIP-1β, VEGF and saliva G-CSF and MIP-1α” (60) in a Ugandan population. In a South African study, the predictive biosignature among the HIV co-infected patients included IFN-gamma, fibrinogen, IFN- alpha-2, MMP-2 (61), and IL-1RA; ApoA-1, CFH, CRP, IFN-g, IP-10, SAA, and transthyretin (62).

#### Whole Blood Culture Cytokine and Chemokine Production

Whole blood assays using recombinant proteins and *Mtb* antigens such as ESAT-6, CFP-10, TB7.7 (63) as well as other recombinant proteins (Rv2029c, Rv2032, Rv2389c) (64) have been evaluated for the prediction of active TB disease. Attempts to translate these promising markers into point of care tests have been made with IP10 and CCL4 that have been incorporated into a lateral flow platform (65). In a multisite African study, unstimulated IFN-gamma, unstimulated TGF-α, and antigen-specific levels of IL-1ra and MIP-1β diagnosed active TB disease with a sensitivity and specificity >70% (48). Among asymptomatic subjects, Apo-A1, ITAC-1, 1-309, MIG, MCP-2, and NCAM-1 in the unstimulated sample (Nil) predicted active TB with a sensitivity and specificity >70%, well as within the TB antigen-stimulated sample, Apo-CIII, I-309, MIP-1α, and TNF-α predicted active TB disease (49).

Despite the advances in this field, the challenge lies in the fact that the most affected participant groups are not well-covered by the testing methods available. These include pediatric participants who cannot produce sputum and the HIV-infected participants who have paucibacillary tuberculosis and usually have negative sputum smear results.

Despite all the research, no new tests based on these bio-signatures currently exist. However, there are studies in advanced stages of development and clinical evaluation utilizing the identified bio-signatures.

##### Mtb RNA for Active PTB Diagnosis

*Mtb* RNA has been used to detect presence of viable bacilli in both sputum culture positive and negative participants. Among the sputum culture negative participants with LTBI, viable but non-culturable bacilli have been retrieved in previous studies having both pulmonary and extra pulmonary TB (66). Attempts to diagnose to active PTB using *Mtb* RNA by analyzing 85B mRNA, IS6110, 16S rRNA and 65 kDa heat shock protein have been made using nucleic acid amplification techniques (67). The IS6110 insertion sequence is being used for active PTB diagnosis for its uniqueness among the *Mtb* complex as a mobile promoter (68) with a role in influencing growth of *Mtb* strains through selective activation of monocytes (69). This has been translated into a quick immune chemical assay with a TAT of 4 h (70). The 16S rRNA gene has been studied for its diagnostic value in *Mtb* complex due to the fact that its highly conserved among *Mycobacteria*. In a South Korean study 16SrRNA provided a sensitivity above 90% using PCR (71). It has also proven to have great value in diagnosis of non-tuberculous mycobacterium (NTMs) although its sensitivity is affected if samples are smear negative (72). Messenger RNA for 85B is used for predicting actively replicating bacilli an important aspect in detecting active PTB disease as well as PTB treatment response (67, 73).

##### Blood Transcriptomic Signatures

Host control and eradication of *Mtb* are directed by the regulation of transcription and host gene expression. These have been highlighted as predictors of active PTB disease or treatment outcomes (26). Tuberculosis gene studies indicated increased expression of genes linked to apoptosis, immune-inflammatory response, bacterial molecular pattern responses (74) regulatory T-cell marker genes, and intracellular trafficking marker genes (74). Alternate splicing of transcripts in blood samples is one of the forms of transcription regulation given that it is also used as an evasion mechanism by *Mtb* to escape immune response (75). Blood gene signatures from paxgene blood samples have proven important for predicting active TB disease. This includes the expression of cytolytic and cell cycle genes which indicates increased lymphocyte proliferation (76–78).

**Figure 2:**
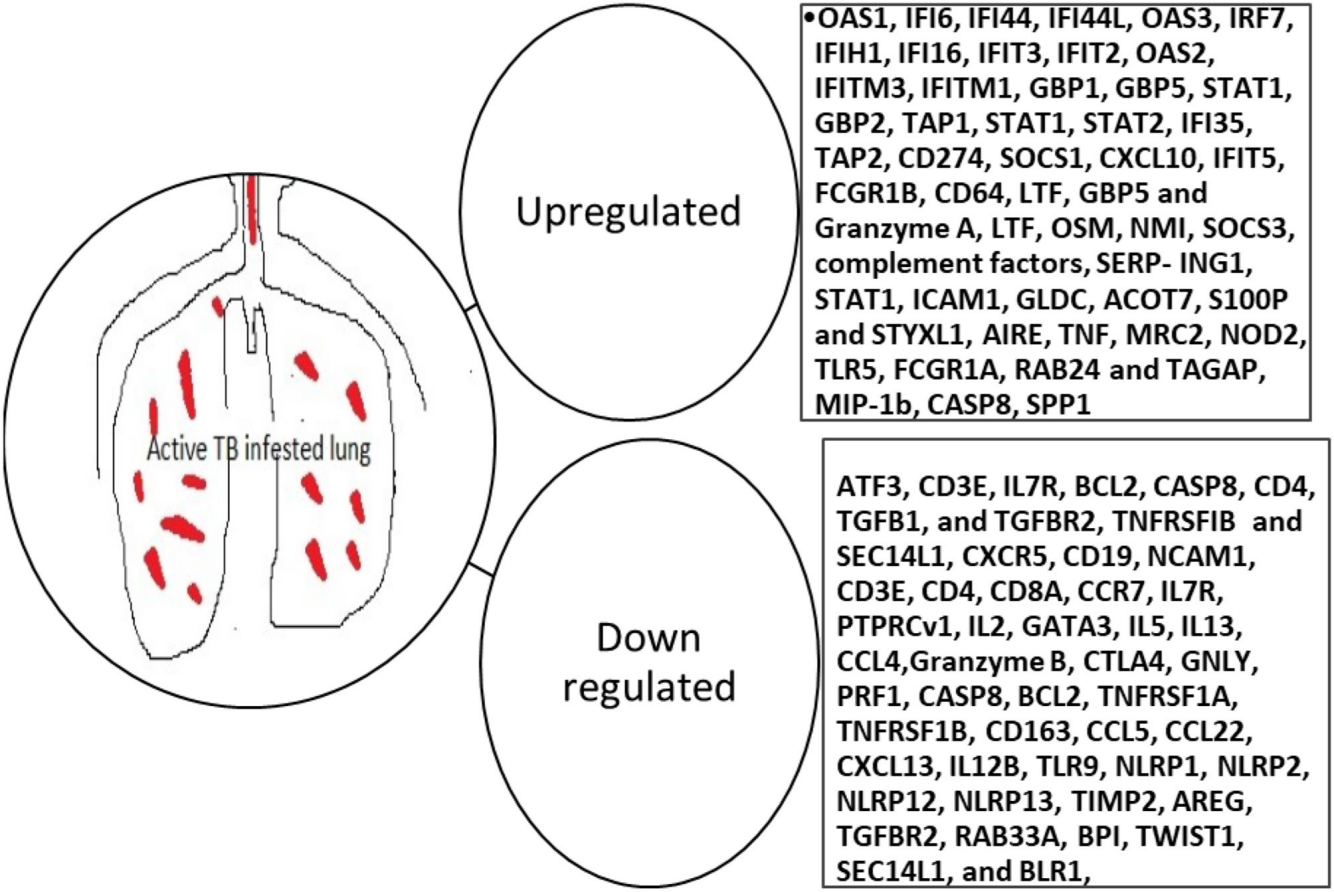
Blood transcriptomic markers predictive of active PTB. OAS, 2'-5'-Oligoadenylate Synthetase 1; IF, Interferon alpha-inducible protein 6; IFI, Interferon Gamma Inducible Protein; IRF, Interferon Regulatory Factor; IFIH, Interferon-induced helicase C domain-containing protein; IFIT, Interferon Induced Protein With Tetratricopeptide Repeats; IFITM, Interferon-induced transmembrane protein; GBP, guanylate-binding protein; STAT, signal transducer and activator of transcription; TAP, Transporter associated with Antigen Processing; SOCS, Suppressor of cytokine signaling; CXCL, C-X-C Motif Chemokine Ligand; FCGR, Low-affinity immunoglobulin gamma Fc region receptor; CD, Cluster of differentiation; LTF, Lactotransferrin; OSM, Oncostatin M; SERP-ING1, plasma protease C1 inhibitor; ICAM, Intercellular Adhesion Molecule; CHRM, Cholinergic Receptor Muscarinic; AMPH, Amphiphysin; SNX, Sorting Nexin; PIGC, Phosphatidylinositol N-acetylglucosaminyltransferase subunit C; TAS2R46, Taste 2 Receptor Member 46; HBD, Hemoglobin subunit delta; GLDC, Glycine Decarboxylase; ACOT, Acyl-CoA thioesterase; S100P, S100 Calcium Binding Protein P; STYXL, Serine/Threonine/Tyrosine Interacting Protein; IL, interleukin; BCL, B-cell lymphoma; CASP, cysteine-dependent aspartate-directed proteases; TGFB, transforming growth factor-beta; TGFBR, transforming growth factor-beta receptor; TNFRSFIB, tumor necrosis factor II receptor; SEC, Selenocysteine; NCAM, Neural Cell Adhesion Molecule; CCR, C-C Motif Chemokine Receptor; PTPRCv, protein tyrosine phosphatase receptor type C; GATA, Erythroid transcription factor. CTLA, Cytotoxic T-Lymphocyte Associated Protein; GNLY, Granulysin; TLR, Toll-like receptor; NLP, Nucleoplasmin-like protein; TIMP, tissue inhibitor of metalloproteinases; AREG, Amphiregulin; RAB, G-protein; BPI, Bactericidal Permeability Increasing Protein; TWIST, Twist-related protein; BLR, beta-lactam resistance protein; AIRE, Autoimmune regulator; MRC, Mannose Receptor C; Nod, nodulation; TAGAP, T-cell activation RhoGTPase activating protein; SPP, Signal Peptide Peptidase.

A blood transcriptional profiling study evaluating the performance of 393 selected transcripts in a high TB endemic setting compared to a low TB setting revealed that these blood transcripts were indicative of successful treatment as they would wane with treatment. Interferon-inducible transcripts were identified and were over-expressed in purified blood neutrophils and to lesser extent monocytes, but not CD4+ and CD8+ T cells, from active TB patients, compared to healthy controls (79). This signature identified by Berry et al. (79) has been cited and utilized by other studies and has proven to be differentiative of disease states as well as treatment response (80–84). These genes include FCGR1B, CD64 (FCGR1A), LTF, GBP5, and GZMA31. Transcriptomic signatures that are predictive of active PTB in recent studies are highlighted in Figure 2. Many of the markers keep coming up in the different studies and therefore ought to be investigated for use in improved diagnosis of active PTB (85).

##### Immune Metabolic Markers

Symptoms of pulmonary tuberculosis such as weight loss and appetite loss are regulated by metabolic alterations in the patients' endocrine system. There is limited data about the use of immune metabolic markers for their use in PTB diagnosis yet they play an important role in the manifestation of TB signs and symptoms.

A study in Italy evaluated plasma endocrine markers like cortisol, dehydroepiandrosterone (DHEA), Leptin, Ghrelin, adiponectin, and pro-inflammatory markers like plasma CRP, IL-1b, IL-6 for their ability to predict PTB among HIV negative PTB patients, their household contacts, and healthy controls. There were significant differences between levels of all the above markers except for DHEA between PTB patients and either household contacts or health controls (86).

Another study in Argentina assessed the use of immune metabolic markers for predicting the efficacy of PTB treatment response. Cortisol and DHEA were assessed at the initiation of treatment, at months 2, 4, and 6 as well as 3 months after treatment completion in this study. Cortisol levels increased during treatment but return to normal by Month 3 post PTB treatment well as DHEA levels were decreased among PTB patients but returned to normal after the end of treatment (87). When comparisons were made, saliva levels of DHEA were reflective of blood levels but not for its sulfated derivative (DHEA-S) (88). Saliva cortisol levels on the other hand were lower than in blood (89, 90).

#### Tuberculosis Detection in Samples Other Than Blood

Globally, there is a push to develop assays that make use of easily available sample types such as urine, saliva as well as the need for assays that do not require overnight culture with the aim of developing rapid and cheaper tests which are possible to implement in resource-limited settings.

Urine lipoarabinomannan (LAM) has been an important marker for TB among the HIV infected (17, 20, 91). Point of care strips for LAM are available on the market for use among the HIV infected. Importantly, the only approved antibody test for TB is the Alere LAM. In the pipeline are the new formats of LAM that are being investigated by FIND; SILVAMP TB LAM (FujiLAM) (92, 93). A study on host markers in urine identified a biosignature consisting of IL2-Ra, sIL2Ra, and MDC (CCL22) that diagnosed active TB disease regardless of HIV status (94).

Saliva use for *Mtb* diagnosis was earlier highlighted (95) and pro-inflammatory and inflammatory markers are in high quantities in saliva (60, 96, 97). Studies comparing serum and saliva levels demonstrated significant differences in the levels of GM-CSF and VEGF in saliva compared to serum (60). Previous studies showed that median levels of IL-17A, IL-23, and ECM-1 were significantly higher in the TB patients and were predictive of treatment response (98). Levels of IL-1β and IL13 were higher in saliva with IL-1β and VEGF appearing among the top markers with diagnostic potential (60, 96) respectively. G-CSF, TNF-α, and VEGF diagnosed active TB in a saliva model among the Ugandan population (60). In a South African population, a biosignature comprising CRP, ferritin, SAP, MCP-1, A2M, fibrinogen, and TPA (97) as well as a signature comprising IL-1β, IL-23, ECM-1, HCC1, and fibrinogen, diagnosed active TB regardless of HIV status. When HIV was put into account, a biosignature comprising, ENA-78, GDF-15, SAA, IL-12(p40), IL-21, IL-13, granzyme A, and PAI-1 diagnosed active TB with a sensitivity and specificity above 90% (98).

Other body fluids that have been used include bronchoalveolar lavage (BAL), breath condensate, and pleural fluid. Breath condensate is still a virgin area of research for *Mtb* diagnosis (20). Bronchoalveolar lavage (6) and pleural fluid are other samples that have been utilized for the diagnosis of active PTB disease because of their proximity to the site of infection. The challenge is the invasive nature of acquiring the sample. Being at the site of infection, BAL provides the true reflection of what happens within the immune system when attacked by *Mtb* (99).

### Effect of Other Diseases on Pulmonary Tuberculosis Disease Diagnosis

“Human immunodeficiency syndrome (HIV)” is a co-infection whose presence fuels progression from latent tuberculosis to active PTB disease. Because HIV compromises the immune system, TST performance, as well as IGRA sensitivity, are highly affected among HIV-infected persons. Lower CD4 counts are associated with higher rates of indeterminate QuantiFERON results. On the other hand, T-SPOT is less affected by HIV immune suppression, as is the QuantiFERON and tuberculin skin test. This is partly because the test utilizes an adequate number of peripheral blood mononuclear cells despite overall low CD4+ cell counts in whole blood (25). For this reason, most studies utilize HIV-negative subjects to avoid confounding results. This is problematic as those infected with HIV are at more risk of progression to active PTB disease compared to the healthy subjects who most studies use. More investigations need to be made in discovering more host-derived biomarker signatures to improve the diagnosis of active PTB in TB endemic areas as well as among the presumptive TB cases that comprise a huge base of TB transmission.

Diabetes has also been shown as another risk factor for progression to active TB disease (100, 101). Another study highlighted diabetes mellitus as a risk factor for tuberculosis but recommended the classification of diabetes mellitus stages for better association studies (102). Interestingly, while diabetes mellitus is a risk factor for tuberculosis, tuberculosis too is a risk factor for the progression of diabetes mellitus (103). An Indian study showed that diabetes mellitus contributed to cavitation during TB disease in either the right or the left lung lobes (104). It is therefore important to assess these and other comorbidities so that they do not become confounders for the results in tuberculosis studies.

## Discussion

### Findings

The diagnosis of PTB remains a challenge globally and enormous research is going on around the globe to improve the currently available PTB diagnostic methods. Immunodiagnostic markers are still not yet approved for PTB diagnosis despite the fact that more research is being made to identify biosignatures that can be incorporated into POCs for PTB diagnosis. Recent advances with the IGRA- T-SPOT assay where the TBAg/PHA ratio has been cited to diagnose active PTB disease. Blood biosignatures that predict active PTB have shown to be divergent in different populations. However, a few have been used to develop POC tests for identifying subjects with active PTB disease thus preventing new infections in the population. Great progress has been made with the nucleic acid amplification techniques for PTB diagnosis and have been used to develop POCs such as the Xpert MTB/RIF assay. More research needs to be made to incorporate other *Mtb* antigens that have proven to have diagnostic potential such as 65 kDa heat shock protein, 85B mRNA, 16S rRNA and IS6110. Host transcriptomic signatures are a promising avenue for use as markers for PTB diagnosis although there's still need for more research regarding biosignature discovery and testing among different populations. This is because, some markers perform better in non PTB endemic areas yet when introduced in endemic areas the results differ a lot. Besides HIV, other illnesses like diabetis mellitus have shown to be risk factors for LTBI progression to active PTB disease. In such cases, subjects who present with these illness may need to be planned for prophylaxis treatment once identified to be having LTBI. In the era of COVID-19 pandemic which presents with symptom similar to those of active PTB, it is important to rule out COVID-19 among patients presenting at the TB clinics to avoid health workers and clinic staff being infected.

### Strengths and Limitations of the Study

Tremendous research in PTB diagnostics has been included in the time frame for this review. This review does not include studies for biomarkers for PTB treatment response, drug resistance and relapse.

## Conclusion and Recommendations

*In vitro* stimulation is not completely representative of immune response to tuberculosis given the doses of recombinant proteins and lack of a regulatory mechanism. In this regard, only parts of the pathogen are used which are quite different from the original pathogen. Whether or not immune cells respond the same way is still an area of great research. Further research ought to be made with the T-SPOT assay in regard to the TBAg/PHA ratio for active PTB diagnosis among PTB endemic areas in large studies. Additionally, molecular diagnostics focusing on *Mtb* RNA markers needs to be spearheaded for POC tests to improve active PTB diagnosis. Subjects with comorbidities that are risk factors for active PTB disease progression need to be intitated on prophylaxis treatment to reduce on the number of new cases as well as TB morbidity and mortality.

Translational research to test and incorporate all investigated markers into point of care active pulmonary tuberculosis tests is a field worth exploring given the available findings. This will provide opportunities for research aimed at reducing active PTB disease diagnostic challenges.

## Author Contributions

AN wrote the original draft. NC and HM-K reviewed the draft for submission. All authors contributed to the article and approved the submitted version.

## Conflict of Interest

The authors declare that the research was conducted in the absence of any commercial or financial relationships that could be construed as a potential conflict of interest.

## Publisher's Note

All claims expressed in this article are solely those of the authors and do not necessarily represent those of their affiliated organizations, or those of the publisher, the editors and the reviewers. Any product that may be evaluated in this article, or claim that may be made by its manufacturer, is not guaranteed or endorsed by the publisher.
